# Study protocol for ‘the effects of multimodal training of cognitive and/or physical functions on cognition and physical fitness of older adults: a cluster randomized controlled trial’

**DOI:** 10.1186/s12877-022-03031-5

**Published:** 2022-05-06

**Authors:** Bik-Chu Chow, Jiao Jiao, David Man, Sonia Lippke

**Affiliations:** 1 Dr. Stephen Hui Research Centre for Physical Recreation and Wellness, Faculty of Social Sciences, Hong Kong Baptist University, Kowloon Tong, Hong Kong, China; 2 Department of Sport, Physical Education and Health, Faculty of Social Sciences, Hong Kong Baptist University, Kowloon Tong, Hong Kong, China; 3 Department of Rehabilitation Sciences, Faculty of Health and Social Sciences, The Hong Kong Polytechnic University, Hung Hom, Hong Kong, China; 4 Department of Psychology & Methods, Jacobs University, Bremen, Germany

**Keywords:** Intervention programs, CRCT, Older adults, Cognitive, Physical training, Behavioral and motivation changes

## Abstract

**Background:**

The elderly population worldwide is increasing exponentially which will be associated with more people suffering from cognition and fitness declines. The well-established benefits of exercise training for the elderly’s cognitive and physical functioning have been observed. However, the amalgamated effect of combining cognitive and physical exercises on the older adults’ cognitive functions, physical fitness, or psycho-related health remains unclear. Thus, this study protocol was planned to conduct different combinations of cognitive and/or physical training interventions to community-dwelling older adults and expected to see the multifaceted effects of the varied combination of training on their health.

**Methods:**

This study is a cluster randomized controlled trial (CRCT). A total of 285 older adults (age ≥ 60) from twenty elderly centres as clusters will be randomly selected and assigned to intervention groups (IGs, *n* = 16) or control groups (CGs, *n* = 4). Each IG will be randomly assigned to one of the four combinations of three training modes that include cognitive (A), physical (B), and combined cognitive and physical training (CCPT, i.e. C), namely Mixed ABC, A + B, C + A, B + C. The intervention will last for 4 months in which the training is conducted for 16 sessions, 2 sessions per week, and 60 min per session. Four repeated assessments (pre-test, two post-training tests after 2 months and 4 months, and a follow-up test) will be conducted. The CG will only receive the four repeated assessments but no intervention. The outcome measures include cognitive tests (tests of execution, memory, and psych-social status), physical fitness, and dynamic balance tests.

**Discussion:**

This study will provide substantial evidence that the integrated format of cognitive and physical exercises training will have higher cognition and fitness impact than the single training modes, and all these mixed modalities will have greater positive outcomes than the control condition. If the effectiveness is proven, the intervention can be further explored and extended to the nation so that many more elderly would be benefited.

**Trial registration:**

The trial has been registered in the ClinicalTrials.gov in U.S. NIH (ID: NCT04727450, date: January 27, 2021).

**Supplementary Information:**

The online version contains supplementary material available at 10.1186/s12877-022-03031-5.

## Background

The aging population worldwide is expected to increase exponentially. The elderly population in Hong Kong is projected to have doubled in size in the coming 20 years, from 16.6% in 2016 to 31.1% in 2036 [1]. Biological aging results in cognitive and physical declines and it contributes to possible loss of functional independence and disability [2], which consequently have profound impacts on public health in terms of healthcare utilization and long-term health cost [3]. Along with the current outbreak of COVID-19, the impacts of social isolation are notable on the older population experiencing detraining and physical inactivity accompanied by a marked decline of mental well-being [4].

In the last decade, researchers have studied brain aging with experimental results revealing the brain being neuroplasticity throughout life [5] and such plasticity can be maintained with regular physical or cognitive stimulation [6]. Numerous observational studies and systematic reviews concluded that various modes of exercises including aerobic exercises, resistance training, the combination of aerobic and resistance, and mind–body exercises (e.g., tai chi, yoga, dance) [5] or cognitive training (e.g., video games, card, and board games) [7, 8], yield positive outcomes in specific cognitive domains. Several evidence has also demonstrated that physical activity can be effective in enhancing mental well-being and preventing mental disorders [9–12]. Based on these exciting pieces of evidence, it can be assumed that the combination of physical and cognitive activities might have more benefits than either intervention individually.

Some researchers have investigated the effect of combined cognitive and physical training (CCPT) using different intervention characteristics [13]. For instance, Shatil (2013) found improvement in cognitive performance in both intervention groups of cognitive training only and CCPT than the control group with 16-week training programs [14]. Chow’s research team has also conducted a pilot study entitled ‘Brain Invigoration Gross Motor Activation Program’ (BIGMAP), which was a CCPT program consisting of a set of 10 cognitive games combined with physical exercises of various difficulty levels [15]. The games and activities of BIGMAP were delivered to 466 elderly people (Mean ± SD: 73.6 ± 7.30 years) from 33 elderly centres in Hong Kong, with each training session lasting for 75 min offered twice a week. After 10 weeks’ training, significant improvements were found in both cognitive and fitness functioning as compared to that at baseline. However, a major weakness of the project was that it was not a randomized controlled trial (RCT), and did not investigate the participant’s motivation and psycho-social related measures. An RCT study is needed to test its effectiveness.

Meanwhile, another research group in Hong Kong conducted a 12-month cluster RCT study focusing on the effectiveness of four different interventions on cognition, which comprised of physical exercise, cognitive activity, CCPT, and social activity for older adults with mild cognitive impairment [16]. They found no changes in dementia stage and mild cognitive impairments tested by Clinical Dementia Rating sum of boxes scores (CDR-SOB) and Disability Assessment for Dementia-Instrumental Activities of Daily Living scores (DAD-IADL) across time and intervention groups. However, a more significant improvement of the Alzheimer’s disease assessment scale-cognitive (ADAS-Cog) subscale, which was considered the gold standard for assessing the efficacy of antidementia treatments, has been observed by post-hoc subgroup analyses after the 12-month CCPT, as compared with the other interventions [16]. A systematic review conducted by Rahe and Kalbe (2015) summarized the findings of four RCTs and four non-RCTs related to the benefits of CCPT on cognition, in which only three out of the four none-RCT studies showed the superiority of combined training when compared to single interventions while all the four RCT studies failed to show any benefit of CCPT [17]. Similarly, the authors in a recent meta-analysis on this research area stated that although the CCPT showed advantage compared to a single intervention, the data is far from being conclusive due to the heterogeneity of studies or too few studies examining the combination effect [18, 19], hence, additional investigation is necessary. Also, according to another two review studies summarizing the possible benefits of CCPT [13, 18], there was a need to determine the effectiveness of CCPT vs. cognitive training only (based on board games) vs. physical training on the cognitive functions, physical fitness, psychosocial measures (i.e. loneliness) of community-dwelling older adults.

### Research objectives and hypothesis

The purpose of this study is twofold: first, to determine the effectiveness of four mixed modalities, namely Mixed ABC, A + B, C + A, and B + C, of three training programs (A: cognitive training, B: physical training, and C: combined cognitive and physical training (CCPT)) on cognition, fitness, and psychosocial-related health of community-dwelling older adults in Hong Kong; and second, to determine which specific training program being most effective among the three programs as compared to the control group (CG).

The hypothesis is that participants with the integrated format of three training modes will have higher cognition and fitness scores than the participants with the other combinations of two training modes, and all these mixed modalities will show greater positive outcomes than the control group.

## Methods

### Research design

**Fig. 1:**
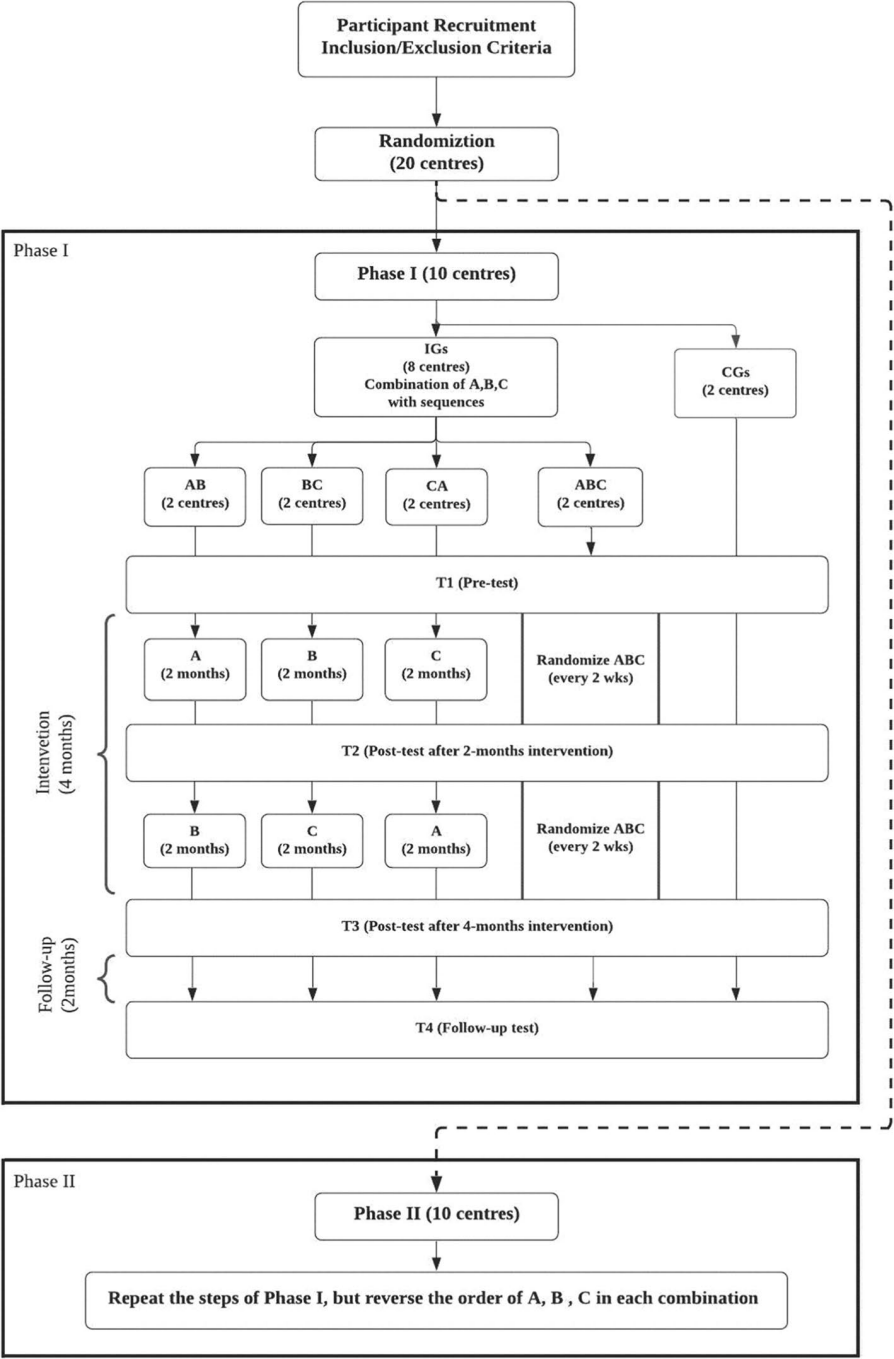
Flow diagram of the study through two phases of the CRCT, including recruitment, allocation, intervention, and data collections points

This study is a cluster randomized controlled trial (CRCT), to be carried out in 20 community elderly centres (see Fig. 1). The elderly centres will be randomly selected from the population list of elderly service providers in the 18 districts of Hong Kong and assigned to either one of the 4 Intervention Groups (IGs) or the Control Group (CG). There will be 16 centres in the IGs and 4 centres in the CG participating in a 6-months program (4-month intervention with 2-month follow-up). The 20 participating centres will be equally allocated to two Phases, I and II, with each Phase involving 8 centres in IGs and 2 centres in CG. Each IG will be randomly assigned to one of the four combinations of three training modes that include cognitive (A), physical (B), and combined cognitive and physical training (CCPT, i.e. C), namely Mixed ABC, A + B, C + A, B + C. The intervention will last for 4 months in which the training is conducted for 16 sessions, 2 sessions per week, and 60 min per session.

In each phase, four repeated assessments (T1-T4) will be conducted: T1 is the pre-test before intervention; T2 and T3 will be conducted on intervention right after the end of 2-month and 4-month, respectively; T4 is a follow-up test conducted 2 months after the completion of all intervention training. Participants of CG will be asked to assume regular-daily habitual activities and take four repeated assessments only.

The study is designed in accordance with the SPIRIT (Standard Protocol Items: Recommendations for Interventional Trials). The study protocol has been approved by University Research Ethics Committee (REC/19–20/0066) and registered at ClinicalTrials.gov Identifier: NCT04727450.

### Sample size

As suggested by Brown et al. (2015) [20], the first step of CRCT sample size estimation is to determine one for a non-clustered design. Based on the repeated measures of ANOVA analyses of 4 trials by 5 groupings (4 IGs + 1 CG) with parameters set to an effect size of 0.175 (small to medium), an alpha level of 0.05, power of 0.90, rho of 0.5, the sample size (*n*_*n*_) estimated is 95 (calculated by G*Power software). Next, the second step is to determine the design effect (*D*_*e*_) for clustering estimation. It is calculated with a group size of 15 and ICC of 0.1 (from previous a study titled BIGMAP, to be published) [15] and yields 2.4. The required sample size for a CRCT is 228 (i.e., *n*_*n*_×*D*_*e*_). By considering a 20% of attrition rate of the participants, the total sample size (*N*) of this study is set at 285, composed of 20 groups/centres of 14–15 participants each.

### Participants recruitment

Eligible elderly participants (age 60 or above) who have passed a cognitive and emotional screening test will be recruited by the community centres with preset inclusion and exclusion criteria. The demographic information of all participants will be collected in terms of age, educational level, medical history, living conditions, occupation, and daily activity patterns. All participants will be informed of the objectives, potential risks, benefits and the data collection procedure of the study. They will sign a written consent prior to their participation.

The inclusion criteria of participants include willingness and capability to give informed consent to participate in the training and testing of this study; and pass the cut-off of the screening tests (cognitive and emotional): Montreal Cognitive Assessment Test (MoCA), a widely used assessment test for cognition [21], with the score above 19 (satisfactory basic function). The elderly will be excluded if s/he has a history of systematic cardiovascular diseases causing cognitive impairments, poorly controlled hypertension, stroke, Parkinson’s disease, early stages of Alzheimer’s disease, brain injury or brain operations, and other complicated conditions that could have prevented attending regular attendance and taking assessments in the elderly centre; concurrent major psychiatric disorder (e.g. major depressive disorder, schizophrenia) or drug and alcohol abuse; and currently receiving regular physical (perform aerobic, flexibility, and stretching exercises for 3 days per week, 45 min per session, for 1 year or longer) and cognitive exercises (such as playing mahjong, chess, bridge, and reading for more than 30 min per session and 3 times per week, for 1 year or longer).

### Interventions

Each selected elderly centre will be randomly assigned to either IGs or CG and asked to help to recruit participants with a sample size of 14–15 older adults. There will be 16 centres in the IGs to receive four combinations of three training modes (A: cognitive training only, B: physical training only, C: CCPT) and with 4 centres in IGs. The four types of combinations are 1. Mixed ABC, 2. A + B, 3. B + C, 4. C + A, matched with 4 CGs in total. For the case of combination 1. Mixed ABC, participants will receive different training modes every 2 consecutive weeks with randomized order. For the case of combinations 2, 3, and 4, the participants’ centres will receive an assigned sequential order of two training modes with each mode lasting for 2 months. All participants of IGs will attend training sessions twice a week, and 60 min duration per session (per mode). The random allocation sequence of intervention was generated by a blinded statistician external to the research team. Due to the nature of each intervention, blinding cannot be applied to the participants, while the blinded group allocation will be applied to the data assessors and analysts. Each subject will be identified by the number code. Data from those participants having more than 70% attendance will be analyzed. No further follow-up with missing cases will be done because they will be usually missing at random (such as absences due to doctor’s appointments or being sick).

The three training modes (A, B, and C) are introduced as follows:

A : Cognitive training only

The cognitive training program will have the participants playing board games in groups. The selection criteria for the board games are: involving a group of 2–4 players, requiring thinking skills in playing, being relatively easy to learn to play and being a fun game. Ten popular commercial board games are selected (Dominos, Yahtzee, Pavilion, Scopa, UNO, Rummikup, Charades, Cranium, Gabblet, Chinese Checker – see Additional file 1 for a brief description). The training format of board games consists of these essential elements as program strategies: having social support, fun, high repetition, and practice, which are deemed to be important by researchers [6] in designing intervention programs. During each intervention session, participants will engage in four randomly assigned games out of the ten games. They will be randomly put in groups with each group engaging in one game for 15 min of playtime. Also, in the last 5 min of each session, participants will be asked to share feedback about the games. An instructor and an assistant recruited from the centre will help to monitor the board games practice in a 60 min session. The elderly centre staff will be trained for 3 h to play and understand the instructions and rules of the board games to be offered to the participants.

B : Physical training only

The physical training program will involve learning three preselected routines of ‘Line dancing’ (see Additional file 2 for a brief description of a sample dance). The current program aims to improve the cardiorespiratory endurance of older adults by learning sequences of walking and turning rhythmic moves with music in a structured group exercise setting. Participants will learn each routine in 5 lessons. The last lesson will be spent on revising all 3 routines. Prior to the start of the study, a panel of three experienced line dancing instructors with experience in teaching older adults will select three-line dancing routines for this program. The panel will help to develop three instructional lesson plans. Moreover, three separate pilot trials of teaching the pre-selected dance routines will be conducted with a group of 10 participants. These participants will wear a Polar heart rate monitor to get heart rate data during training at pilot trials so that the moderate-to-vigorous intensity levels and the targeted heart rate zone (according to ACSM guidelines on exercising intensity for older adults [22]) can be documented. The format of training in a session will include 10 min warm-up, 45 min main training, and 5 min cool-down. One major instructor with one assistant will lead the prescribed activities for a ratio of 1 leader to 7–8 older adults to ensure adequate instruction and supervision of the participants. ‘Line dancing’ certified instructors will be recruited either from the Hong Kong Line Dancing Association, other dance associations, or through media. The ‘Line dancing’ instructor has to attend a 2-h refresher course on the instructional lesson plans of the three preselected line dance routines.

C : Combined cognitive and physical training (CCPT)

This program will adopt the BIGMAP which was trialed out with older adults in the community elderly centres by data collection of nonverbal Stroop test on cognition, functional fitness test based on senior fitness test and heart rate data. This program provided a set of ten cognitive games combined with physical training with various difficulty levels [5] designed by cognitive psychologists and exercise scientists. Each game is targeted to improve on cognitive functions by training on visual, spatial, hearing memory, and proprioception as well as physical fitness functions through physical and motor skills training on moderate-intensity aerobics activities, muscular endurance, balance, reaction, and coordination [15] (see Additional file 3 for descriptions of BIGMAP). Based on empirical evidence, BIGMAP involves moderate intensity of aerobics training (mostly of walking activities) to the participating older adults. One major instructor with two assistants will lead the prescribed BIGMAP activities (including physical training and cognitive games) for a ratio of 1 leader to 5 older adults to ensure adequate instruction of the participants. A three-hour off-site classroom training and one-hour on-site training will be offered by an experienced supervisor involved with previous BIGMAP study to the instructor and assistant of the CCPT mode following the training manual of BIGMAP [15].

In regards to the monitoring of the instructors’ quality, a supervision procedure will be applied to all training modes that is having an experienced trainer observing the first session of an intervention program to ensure proper instruction in both phases of the study. Therefore, the instruction quality of each training mode will be affirmed.

### Outcome measures

Participants will take part in evaluation for four times, including a pre-test (T1), two post-tests (T2 and T3) at 2-month intervals, and a follow-up test (T4) two months after the intervention. The evaluators will be blind to the group membership of participants. Each evaluation includes three aspects of testing that are cognitive tests (60 min), fitness tests (30 min), and mental-related surveys (15-20 min). The total testing time per participant is less than 2 h.

#### Primary outcome: cognitive measures

The main cognitive outcome measures will target executive functions which involve a variety of higher-order cognitive processes, such as cognitive flexibility, planning, decision making, attention, and memory. Three tests, Wisconsin Card Sorting Test-computer version (WCST), Color Trial Test (CTT), and Rivermead Behavioral Memory Test (RBMT) will be used as the cognitive outcome measures. These tests are chosen based on their relevance to the BIGMAP contents which were mainly designed to train cognition in terms of executive functions and memory, and physical functions. These well-established tests with proven validity and reliability are all commonly used in studies testing cognitive functions [20, 23–25], and all the tests have been translated into Chinese used in Hong Kong. Details of test descriptions are listed as follow:

The WCST is one well-known measure of executive functions, which involves mainly the assessment of abilities related to shifting cognitive strategies and abstract reasoning in response to environmental contingencies [26]. Before testing, color blindness needs to be excluded so that it does not influence the test result [27]. A computer version of WCST with 128 cards will be used in this study [28]. The participants will be required to sort two sets of 64 responses cards to match either number (one, two, three, and four), form (circles, crosses, stars, and triangles), or color (red, yellow, green, and blue), which will discreetly and periodically change during the course of sorting without the participants being informed. The participants have to shift sets accordingly and sort cards following the new sorting rule in a computer. Set shifting difficulties will be indicated by preservative errors; thus, higher scores on this test represent worse performance [29, 30]. The WCST is scored according to nine categories and will be automatically summarized in a result sheet provided by the computer. It takes approximately 30 min per participant to complete this test.

The Chinese CTT will be selected to assess the sustained attention and divided attention of the participants as it requires cognitive flexibility and visuomotor skills to complete the task. The CTT is not a language biased test but relies on the use of numbered, colored circles and sign language symbols, thus it is suitable for elderly participants no matter whether they can read English alphabet letters or Chinese characters. The CTT is comprised of two parts: (a) CTT1 is administered first and requires the participant to connect 25 number circles, which are randomly located on a sheet of paper, in an ascending numbered sequence (1 to 25); (b) In CTT2, the participant is required to connect number circles from 1 to 25 while alternating between two colors—pink and yellow. In this study, the time-related indices, such as Time of CTT1 and CTT2 (in seconds), CTT2-1 time difference, etc. will be recorded and used for statistical analysis [31, 32].

The Chinese RBMT will be used for the assessment of participants’ memory in this study [33]. It consists of 11 subtests that cover the aspects of visual, verbal, recall, recognition, immediate, and delayed every memory. Additionally, prospective memory skills and the ability to learn new information are also measured. It will take around 30 min to complete the testing. Raw scores of the 11 subtests will be converted into subtest scaled scores. The sum of 11 scaled scores will be recorded. In addition, a General Memory Index (GMI), representing overall memory performance is also created based on the sum of scaled scores and a conversion table. From the conversion tables, the confidence intervals and percentile ranks for each GMI will be also reported [34].

#### Secondary outcome: physical fitness measures

The fitness outcome measures involve the measures of height, weight, body composition, and senior physical fitness test battery [35]. Four senior physical fitness tests including seat-chair stand, arm curl, 2-min step test, and 8-feet up-&-go will be adopted except for back scratch and chair sit-&-reach. Flexibility will not be tested because both CCPT and physical training programs are not designed for flexibility training. One additional test of the ‘four-square step test’ on dynamic balance assessment [36] will be taken because CCPT and physical training programs involve balance training. In particular, the Line dancing routines involve many walking and body turning movements, which require dynamic balance. All the selected tests have been previously found good reliability and validity when testing older adults [35]. These five tests have been used to assess fitness and balance functions in cognitive or exercise intervention studies [37–39]. The functional fitness norms have been established for community-dwelling older adults in Hong Kong [40].

#### Other pre-specified outcomes: psycho-social-related measures

Scales of behavior changes, intention/motivation, physical activities, loneliness physical health, infection with coronavirus, and subjective health status will be measured in one survey (see Additional file 4). The Centre for Epidemiological Studies-Depression (CES-D) will be used to measure how often over the past week the participants experienced symptoms associated with depression, such as poor appetite, feeling lonely, and restless sheep [41]. The score of CES-D is the sum of the 20-items with a possible range of 0 to 60 in total. The scoring of positive items is reversed, which means the higher scores indicate the presence of more symptomatology. The participants who obtain a score of 16 or greater are considered at risk of depression. If more than 4 questions have missing answers, the score of CES-D will not be marked. All the questionnaires are administered using the Qualtrics system.

### Statistical analysis plan

Data analysis will be performed using the Statistical Package for social sciences (IBM SPSS ver. 27). All data points that were considered outliers, which are out of the interquartile range (IQR) covering the 50% central of samples, will be excluded from analyses. For analyzing program effects, a mixed-effects ANOVA will be used. In this model, there will be a between-subjects factor consisting of five groups (CGs = 0, IGs = 1 to 4) and a within-subject factor consisting of four Trials (Time = 1 to 4). The dependent variables will be the outcome measures of the three cognitive tests and the five physical fitness tests. The independent variables will be time (1 pre-test, 2 post-tests, and 1 follow-up) and intervention (IGs with 4 combinations of training modes & CGs). The participating elderly centre as a cluster will be treated as a random effect. Assumptions (normality, sphericity, etc.) for mixed-effects ANOVA will be tested using an alpha level set as 0.05. The focus of this mixed-effects ANOVA will be mainly on the between-group differences, i.e., the differences among the four IGs. Post-hoc comparisons among IGs will be conducted with Bonferroni correction after finding significant effects. Next, for analyzing specific intervention effects in the 3 treatments crossover design, a similar mixed-effects ANOVA will be used. In this second model, there will be one within-subject factor of four repeated trials (Time = 1 to 4). The dependent variables will be the outcome measures of the three cognitive tests and the four physical fitness tests. The independent variables of time (pre-test, 2 post-tests, and follow-up) and three training modes will be examined by ANOVA with repeated measures. The focus of the second model is to examine the individual effect of the three training modes, so a pre-planned contrast will be conducted to examine their effects. Based on these statistical analyses, results and conclusions will be drawn.

## Discussion

The elderly population worldwide is expected to increase exponentially. There will be a higher percentage of elder adults increasing in Hong Kong in the coming decades which will be associated with more population suffering from cognitive and fitness decline. The growing aging population will also have a significant impact on the public finances including social healthcare costs and medical expenses. Thus, it is necessary to conduct programs to postpone the functional decline and promote the concept of ‘healthy aging’ among the elderly community. From the previous literature, the benefits of cognitive and/or physical exercise training have been well documented. However, how a combination of these training might benefit the cognitive functions, physical fitness, psycho-related health of community-dwelling older adults in Hong Kong remains unclear. This study takes a holistic approach to provide a detailed account of the implementation and evaluation of cognitive and/or fitness training intervention and is expected to see the multifaceted effects of the varied combination of training modes on the elderly’s health.

If the intervention programs would be proven to be effective, the immediate impact will be those community-dwelling elder adults joining the programs would not only increase cognitive and physical health but also provide practice models to develop similar structural programs providing elderly populations with psychosocial support. The concept of ‘healthy aging’ could be promoted to the general population by exposing the study to publicity. The program used in this study can be further explored to be promoted to other sectors of society so that a higher number of older adults can benefit from the program. The results can provide further evidence for the government departments including the Elderly Commission in Hong Kong or other overseas regions to implement new social policy, such as allocating more financial support for the non-government organization to set up local training centres to offer effective cognitive-cum-physical game-oriented activities. Therefore, the number of older adults being frail and having early loss of functional independence and disability will be decreased due to a reduction of the percentage of them having a cognitive and physical decline.

These significant impacts will transfer to a reduction in the public healthcare cost associated with frailty, cognitive and mental problems in the aging society of Hong Kong and worldwide. When more elderly populations consistently engage in the training for several years, they would realize the importance of self-responsibility and a healthy lifestyle in their own health maintenance, which would be a long-term impact on human society.

## Supplementary Information

**Additional file 1.**

**Additional file 2.**

**Additional file 3.**

**Additional file 4.**

## Data Availability

Not applicable.

## Abbreviations

ACSM: The American College of Sports Medicine
RCT: Randomized controlled trial
WHO: World Health Organization
NIH: National Institutes of Health
SPIRIT: Standard Protocol Items: Recommendations for Interventional Trials
BIGMAP: Brain Invigoration Gross Motor Activation Program
BMI: Body mass index
IG: Intervention group
CG: Control group
A: Cognitive training
B: Physical training
CCPT: Combined cognitive and physical training
REC: Research Ethics Committee
SD: Standard deviation
MoCA: Montreal cognitive assessment test
CES-D: The center for epidemiological studies-depression
CTT: Color trial test
WCST: Wisconsin card sorting test
RBMT: Rivermead behavioral memory test
ANOVA: Analysis of variance
