# Supplementary material for: Study protocol for ‘the effects of multimodal training of cognitive and/or physical functions on cognition and physical fitness of older adults: a cluster randomized controlled trial’
Source: BMC Geriatr. 2022 May 6;22:398. doi: 10.1186/s12877-022-03031-5 (PMC9073468; doi:10.1186/s12877-022-03031-5)
Supplement: Supplementary file 1 — Additional file 1. [file 12877_2022_3031_MOESM1_ESM.docx]

**Additional file 1. Cognitive Training (Board Games)**

| Intervention Characteristics | 16 wk, 2 d/wk, 60 min/session  Participants will be assigned into 4 groups and play 1-2 assigned games for 30 min. | | |
| --- | --- | --- | --- |
| Board Games Characteristics | 10 Fun games requiring memory, skill, and strategy (4 selected samples of different difficulties below) | | |
| Game 1: Dominos | Game 2: Yahtzee | Game 3: Uno | Game 4: Rummikub |
| 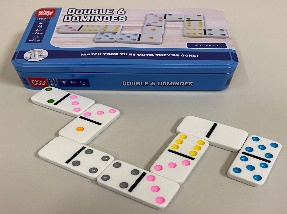 | 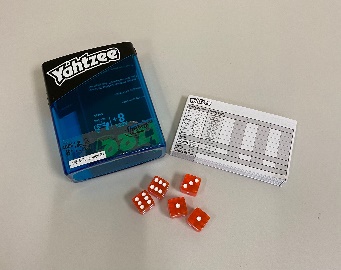 | 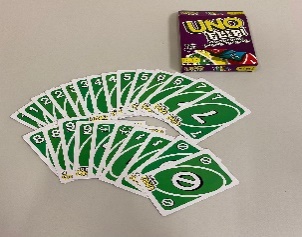 | 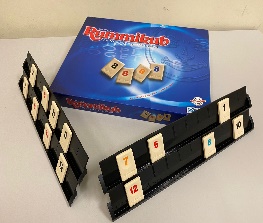 |
