# Supplementary material for: Study protocol for ‘the effects of multimodal training of cognitive and/or physical functions on cognition and physical fitness of older adults: a cluster randomized controlled trial’
Source: BMC Geriatr. 2022 May 6;22:398. doi: 10.1186/s12877-022-03031-5 (PMC9073468; doi:10.1186/s12877-022-03031-5)
Supplement: Supplementary file 2 — Additional file 2. [file 12877_2022_3031_MOESM2_ESM.docx]

**Additional file 2. Physical Training (Line Dancing Routines)**

| Intervention Characteristics | 16 wk, 2 d/wk, 60 min/session Participants will learn a total of 3 dance routines. |
| --- | --- |
| Line-Dancing Characteristics | 3 Dancing routines of moderate intensities (40-60 %MHR) focusing on cardiorespiratory fitness and dynamic balance |
| Sample Dance | [Macarena Dance](https://dance.lovetoknow.com/Macarena_Dance) |
