# Supplementary material for: Study protocol for ‘the effects of multimodal training of cognitive and/or physical functions on cognition and physical fitness of older adults: a cluster randomized controlled trial’
Source: BMC Geriatr. 2022 May 6;22:398. doi: 10.1186/s12877-022-03031-5 (PMC9073468; doi:10.1186/s12877-022-03031-5)
Supplement: Supplementary file 3 — Additional file 3. [file 12877_2022_3031_MOESM3_ESM.docx]

**Additional file 3. Combined Cognitive and Physical Training-CCPT (BIGMAP)** [[15](#_ENREF_15)]

| BIGMAP  Intervention Characteristics | 16 wk, 2 d/wk, 60 min/session Participants will be assigned into 3 groups and practice a task for 15 min. | | |
| --- | --- | --- | --- |
| BIGMAP Characteristics | 10 “Think and Act” tasks with various levels of difficulties involving low to high intensities of mental and physical activities. | | |
| Sample Task | Square Walking consists of 12 different levels. | | |
| 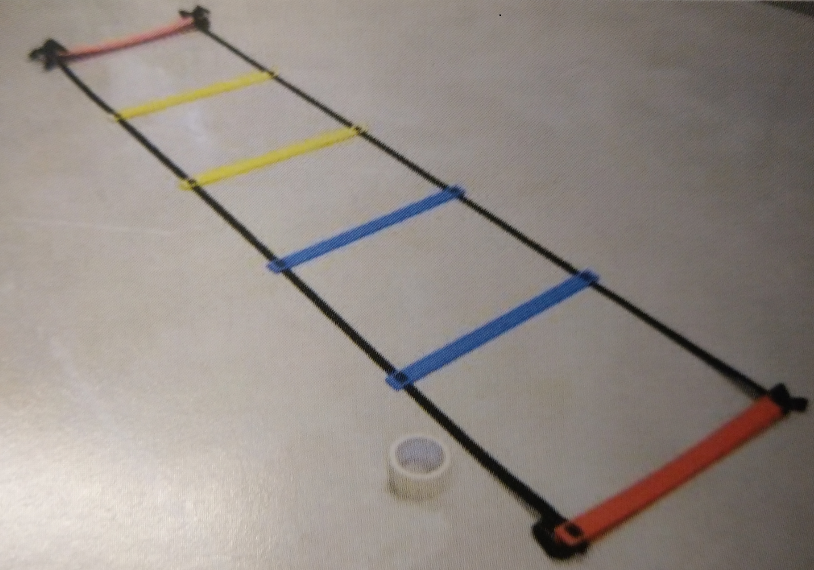  Red=Left foot  Blue=Right foot  1…20 Step sequence | Level 1 Walk Forward | Level 4 Side Stepping | Level 12 Cross Stepping |
|  | 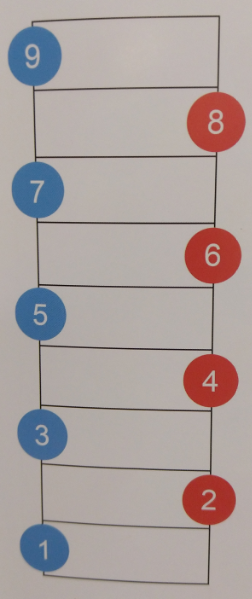 | 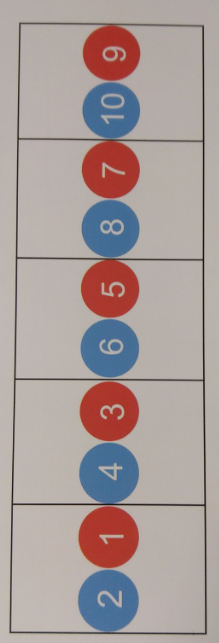 | 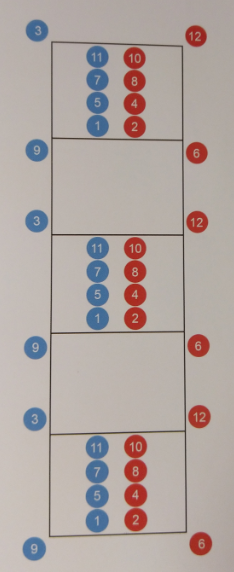 |
