# Supplementary material for: Study protocol for ‘the effects of multimodal training of cognitive and/or physical functions on cognition and physical fitness of older adults: a cluster randomized controlled trial’
Source: BMC Geriatr. 2022 May 6;22:398. doi: 10.1186/s12877-022-03031-5 (PMC9073468; doi:10.1186/s12877-022-03031-5)
Supplement: Supplementary file 4 — Additional file 4. [file 12877_2022_3031_MOESM4_ESM.docx]

**Additional file 4. Psycho-social-related Questionnaires**

**Behavior Changes Questionnaire**

| The Leisure and Cultural Services Department have recommended the citizens to conduct at least 150 minutes (2.5hours) of **moderate exercise**, or at least 3 days of muscle training every week.  **Moderate-intensity activity is usually made up of exercises that get your heart rate up, breathing fast or sweating slightly.** | | | | |
| --- | --- | --- | --- | --- |
| **Behavior alternative** | | | | |
| 1. Choose one of the four statements below that best describes your level of physical activity (over the past three months). [42] | | | | |
| ☐ I am never/ less than one time per month physically active.  ☐ I engage in some light physical activities but not up to 150 minutes a week  ☐ I engage in physical activities regularly but not up to 150 minutes per week.  ☐ I engage in physical activity at least 150 minutes a week (on 5 days per week for 30 minutes or more). | | | | |
| 2. How often are you currently physically active for 30 minutes or more? [42] | | | | |
| ☐ Never or less than 30 minutes a week ☐ Once a week ☐ Twice a week  ☐ Three or four times a week ☐ More than five times a week | | | | |
| **Behavior and intention/motivation** | | | | |
| 3. Compared to before the onset of the Coronavirus pandemic: Have you become less or more physically active? [42] | | | | |
| ☐ I've become less physically active  ☐ About the same  ☐ I'm active more often or for longer | | | | |
| 4. Did you engage in physical activities at least for 150 minutes during the week, in such a way that you are moderately exhausted (e.g., brisk walking, bicycling or swimming)? [42] | | | | |
| ☐ No, and I do not intend to start  ☐ No, but I am considering it  ☐ No, but I seriously intend to start  ☐ Yes, but only during the outbreak of COVID-19  ☐ Yes, and for a long time before the outbreak of COVID-19 | | | | |
| 5. Did you do muscle strength training two or more times a week (e.g., back, leg or abs exercises)? [42] | | | | |
| ☐ No, and I do not intend to start  ☐ No, but I am considering it  ☐ No, but I seriously intend to start  ☐ Yes, but only during the outbreak of COVID-19  ☐ Yes, and for a long time before the outbreak of COVID-19 | | | | |
| **Loneliness** | | | | |
| 6. How often are you unhappy being withdrawn? [43] | | | | |
| ☐ Never | ☐ Rarely | ☐ Sometimes | ☐ Often |  |
| 7. How often do you feel being neglected？[43] | | | | |
| ☐ Never | ☐ Rarely | ☐ Sometimes | ☐ Often |  |
| 8. How often do you feel lonely? [41] | | | | |
| ☐ Never | ☐ Rarely | ☐ Sometimes | ☐ Often |  |
| **Physical health** | | | | |
| 9. Do you have any chronic diseases such as heart disease, diabetes, cancer, respiratory illnesses, liver or kidney disease? [44] | | | | |
| ☐ Yes | ☐ No |  |  |  |
| **Infection with coronavirus** | | | | |
| 10. Have you been infected with the Coronavirus? [44] | | | | |
| ☐ Definitely not, the test was negative  ☐ I don't know  ☐ Definitely yes, the test was positive | | | | |
| **Subjective health status** | | | | |
| 11. How would you rate your health in general? [45] | | | | |
| ☐ Poor | ☐ Fair | ☐ Good | ☐ Very good | ☐ Excellent |
